# Supplementary material for: Fusion and expansion of vitellogenin vesicles during Caenorhabditis elegans intestinal senescence
Source: Aging Cell. 2022 Oct 5;21(11):e13719. doi: 10.1111/acel.13719 (PMC9649609; doi:10.1111/acel.13719)
Supplement: Supplementary file 3 — Figure S1–S3 [file ACEL-21-e13719-s001.pdf]

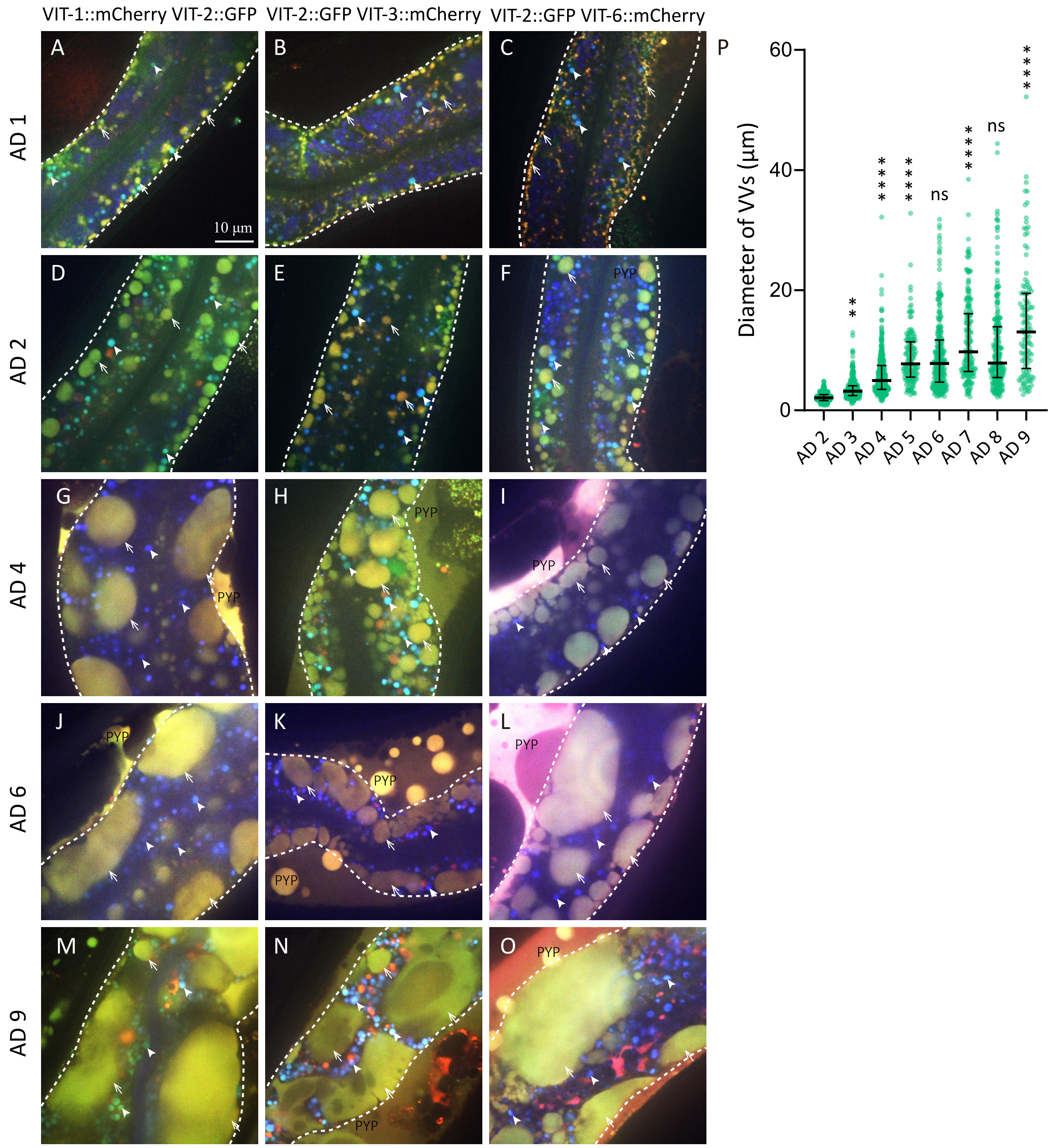

Figure S1. The size of VV increases dramatically with age seen by fluorescent microscopy

White arrows point to VVs, and arrowheads indicate gut granules. White dashed lines outline the intestine. PYP means pseudocoelomic yolk patch. (P) Quantification of the diameters of VVs based on fluorescent and DIC merged micrographs of *vit-1::mCherry vit-2::gfp* KI worms of different ages. 11, 11, 22, 15, 27, 24, 24, 22 micrographs were quantified for AD 2, 3, 4, 5, 6, 7, 8, 9, respectively. Median and the interquartile range are indicated. ****$p < 0.0001$; **$p < 0.01$; ns: not significant; all compared to the former day; one-way ANOVA with Tukey's multiple comparisons test was performed to compare each two data sets.

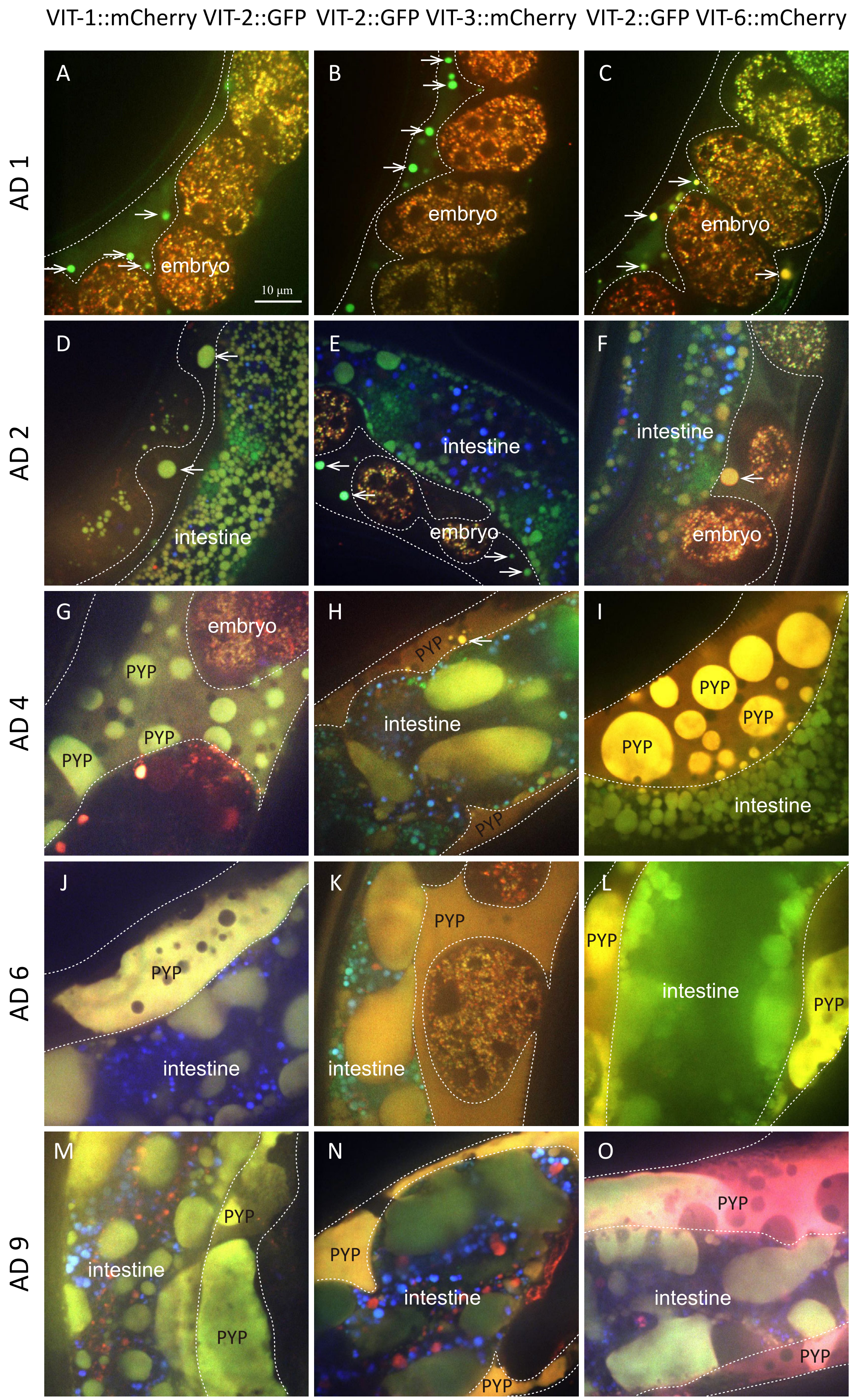

Figure S2. Yolk substances accumulate in the pseudocoelom with age seen by fluorescent microscopy.

(A-O) White dots outline the pseudocoelomic region that can be recognized in epifluorescent images. White arrows and PYPs indicate pseudocoelomic yolk patches.

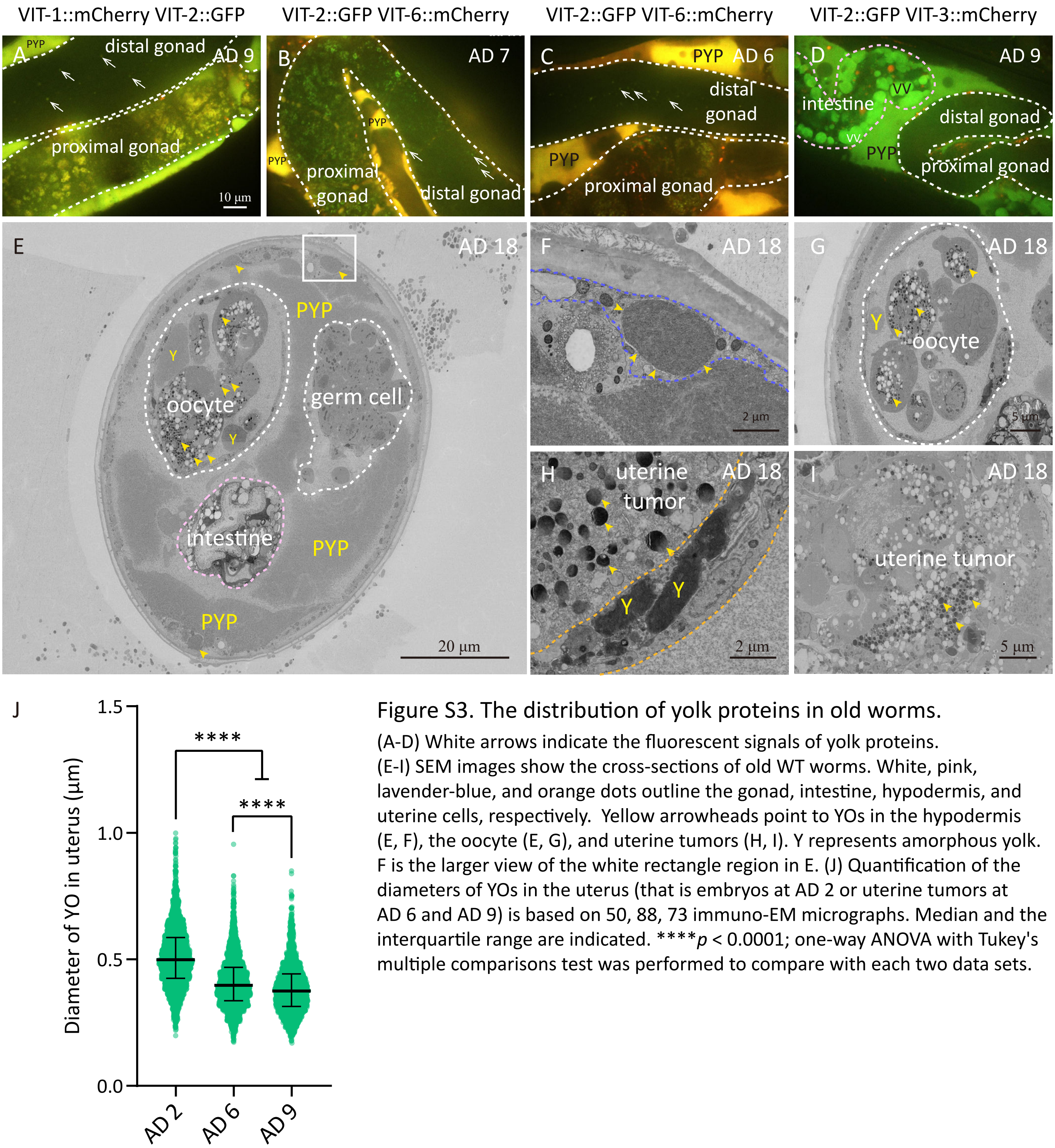

Figure S3. The distribution of yolk proteins in old worms.

(A-D) White arrows indicate the fluorescent signals of yolk proteins.
(E-I) SEM images show the cross-sections of old WT worms. White, pink, lavender-blue, and orange dots outline the gonad, intestine, hypodermis, and uterine cells, respectively. Yellow arrowheads point to YOs in the hypodermis (E, F), the oocyte (E, G), and uterine tumors (H, I). Y represents amorphous yolk. F is the larger view of the white rectangle region in E. (J) Quantification of the diameters of YOs in the uterus (that is embryos at AD 2 or uterine tumors at AD 6 and AD 9) is based on 50, 88, 73 immuno-EM micrographs. Median and the interquartile range are indicated. ****$p < 0.0001$; one-way ANOVA with Tukey's multiple comparisons test was performed to compare with each two data sets.
